# Slow Turnover of HIV-1 Receptors on Quiescent CD4^+^ T Cells Causes Prolonged Surface Retention of gp120 Immune Complexes *In Vivo*

**DOI:** 10.1371/journal.pone.0086479

**Published:** 2014-02-06

**Authors:** Yasuhiro Suzuki, Hiroyuki Gatanaga, Natsuo Tachikawa, Shinichi Oka

**Affiliations:** 1 The Department of Infectious Diseases, Graduate School of Medicine, Tohoku University, Sendai, Japan; 2 AIDS Clinical Center, National Center for Global Health and Medicine, Tokyo, Japan; 3 The Department of Infectious Diseases, Yokohama Municipal Citizen’s Hospital, Yokohama, Japan; New York University, United States of America

## Abstract

Peripheral blood CD4^+^ T cells in HIV-1^+^ patients are coated with Ig. However, the causes and consequences of the presence of Ig^+^ CD4^+^ T cells remain unknown. Previous studies have demonstrated the rapid turnover of viral receptors (VRs) on lymphoma and tumor cells. The present study investigates the turnover of VRs on peripheral quiescent CD4^+^ T cells (qCD4s), which are the most abundant peripheral blood CD4^+^ T cells. Utilizing pharmacological and immunological approaches, we found that the turnover of VRs on qCD4s is extremely slow. As a result, exposure to gp120 or HIV-1 virions *in vitro* causes gp120 to remain on the surface for a long period of time. It requires approximately three days for cell-bound gp120 on the surface to be reduced by 50%. In the presence of patient serum, gp120 forms surface immune complexes (ICs) that are also retained for a long time. Indeed, when examining the percentages of Ig^+^ CD4^+^ T cells at different stages of HIV-1 infection, approximately 70% of peripheral resting CD4^+^ T cells (rCD4s) were coated with surface VRs bound to slow-turnover gp120-Ig. The levels of circulating ICs in patient serum were insufficient to form surface ICs on qCD4s, suggesting that surface ICs on qCD4s require much higher concentrations of HIV-1 exposure such as might be found in lymph nodes. In the presence of macrophages, Ig^+^ CD4^+^ T cells generated *in vitro* or directly isolated from HIV-1^+^ patients were ultimately phagocytosed. Similarly, the frequencies and percentages of Ig^+^ rCD4s were significantly increased in an HIV-1^+^ patient after splenectomy, indicating that Ig^+^ rCD4s might be removed from circulation and that non-neutralizing anti-envelope antibodies could play a detrimental role in HIV-1 pathogenesis. These findings provide novel insights for vaccine development and a rationale for using Ig^+^ rCD4 levels as an independent clinical marker.

## Introduction

The most immunogenic HIV-1 molecules for the elicitation of an antibody (Ab) response appear to be envelope (env) glycoproteins, and high titers of anti-gp120 and anti-gp41 Abs are observed in HIV-1-infected patients (HIV-1^+^ Pts) [1]–[3]. However, it is apparent that the neutralizing Ab response in infected patients is weak compared with non-neutralizing HIV Abs [4]. Therefore, non-neutralizing Abs are dominant in the circulation of HIV-1^+^ Pts. Nevertheless, the role of non-neutralizing anti-env Abs in HIV-1 infection remains unclear. More than 95% of the body’s CD4^+^ T cells reside in lymphoid tissues, which are the major sites for HIV-1 replication, CD4^+^ T cell depletion [5], and development of anti-env Ab-secreting B cells [3], [6]. CD4^+^ T cells continuously travel between the blood, the lymphatic system, and lymph nodes (LNs) and re-circulate into the blood over a period of approximately 1 d [7]–[9]; therefore, most peripheral blood CD4^+^ T cells are recent emigrants from the LNs. Because a large proportion of HIV-1 is produced in the LNs (10^10^–10^11^ virions/d) [7], [10]–[14], it is assumed that target CD4^+^ T cells in LNs are continuously exposed to high concentrations of HIV-1 as well as anti-env Abs. In the presence of HIV-1^+^ Pt serum, gp120 forms surface immune complexes (sICs) on HIV-1-infected cells or uninfected cells coated with gp120 *in vitro* [15]. Natural killer (NK) cells have been shown to be able to eliminate gp120/HIV-1-coated or HIV-infected target cells by Ab-dependent cell-mediated cytotoxicity (ADCC) [1], [15]–[20]. However, compared with the distribution in non-lymphoid organs, a relatively small number of NK cells are present in the LNs [21]; therefore, the organs where sICs appear to form on target cells and the effector cells that can eliminate sIC^+^ cells seem to be segregated *in vivo*.

For practical reasons, the dynamics of viral receptors (VRs) and cell-bound gp120/HIV-1 have been extensively studied in both lymphoma and VR-transfected cancer cells. The cell-surface CXCR4 receptors on lymphoma [22], [23] and HeLa cells [22]–[24] are rapidly internalized, and approximately 100% of the cell-surface CXCR4 pools are exchanged every 5 h (in lymphoma cell lines) and 40 min (in HeLa cells). Moreover, cell-bound gp120 has been shown to be internalized in 2 h in Jurkat cells [25], 1 h in CD4-transfected HeLa cells [23], [24], and 1–2 h in U937 cells [24]; therefore, the gp120-VR complex is believed to be rapidly removed from the surface of target cells. Consequently, even if gp120/HIV-1-VR complexes form on CD4^+^ T cells *in vivo*, it has been thought that the complex would disappear from the cell surface before encountering ADCC effector cells. Collectively, it is believed that cell-bound gp120 or HIV-1 on VRs on CD4^+^ T cells have a limited effect on the destruction of HIV-1-exposed cells *in vivo*. In contrast, substantial percentages of CD4^+^ T cells in HIV-1^+^ Pts are shown to be coated with Ig [26], [27]. Because the gp120-VR complex was thought to be rapidly removed from the cell surface, it was also believed that sICs on CD4^+^ T cells in HIV-1^+^ Pts mainly reflect the non-specific attachment of Ig-virion complexes (known as circulating immune complexes; cICs) in serum to the cellular surface [28].

The most abundant HIV-1 target cells *in vivo* are quiescent CD4^+^ T cells (qCD4s) because they comprise more than 90% of both peripheral and lymphoid T cells [14], [29]. However, the dynamics of cell-surface molecules on quiescent cells are generally shown to be slower than on cancer or activated cells [30]. Furthermore, qCD4s have been shown to have unique biological characteristics, particularly the possession of static cortical actin barriers [31], [32] and abundant expression of SAMHD1, a deoxynucleoside triphosphate triphosphohydrolase, to prevent reverse transcription of HIV-1 RNA [33].

Here, we first reevaluated the turnover dynamics of VRs in qCD4s compared with lymphoma cells. We then examined the dynamics of cell-bound gp120 in qCD4s. gp120/HIV-1-exposed qCD4s were further exposed to anti-env Abs to form sICs and to examine their pathological effects. We also investigated the characteristics of sICs on CD4^+^ T cells purified from HIV-1-infected Pts and conducted a longitudinal analysis of the changing levels of sIC^+^ CD4^+^ T cells in peripheral blood from HIV-1^+^ Pts under various conditions.

## Results

### Slow Turnover of VRs in Dense Resting CD4^+^ T Cells

We first thoroughly reevaluated the turnover dynamics of VRs and cell-bound gp120 or HIV-1 on qCD4s by employing highly purified dense resting CD4^+^ T cells (drCD4s) from healthy donors. drCD4s are purified from resting CD4^+^ T cells as a dense fraction using discontinuous density gradients of Percoll (see **Materials and Methods**) [34]. We have previously shown that these drCD4s are largely in the G_0_ phase of the cell cycle, do not produce detectable cytokines, and are highly resistant to spontaneous cell death; therefore, drCD4s are a useful tool for observing biological responses over a long period while avoiding a decrease in viability and spontaneous cell activation in cell culture [34].

**Figure 1:**
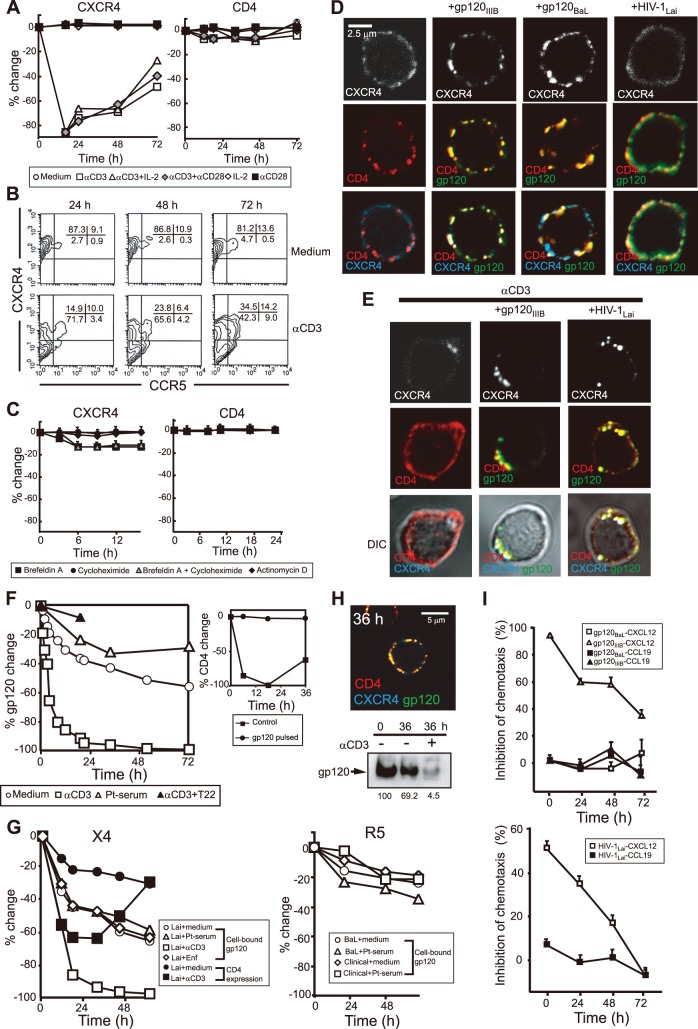
HIV-1/gp120 remains on the surface of qCD4s for a long period of time due to slow VR turnover. (**a, b**) Time course of surface VR expression (**a**) and representative FACS of CXCR4/CCR5 expression on qCD4s (**b**) following a variety of activation stimuli. (**c**) The effect of BFA (10 µg/ml), cycloheximide (50 µg/ml) and ActD (20 µg/ml) on the surface expression of CXCR4 (left) and CD4 (right) on qCD4s. (**d, e**) Confocal micrographs of CD4, CXCR4, and gp120 in qCD4s that were exposed or not exposed to the indicated strain of gp120 or HIV-1 before (**d**) or after (**e**) 16 h of anti-CD3 Ab exposure. qCD4s with (**e**) or without (**d**) permeabilization were stained with anti-CD4 goat polyclonal Abs (Cy3, red), anti-CXCR4 mouse mAbs (Qdot 655, blue), and anti-gp120 rabbit antiserum (Cy2, green). (**f**) Time course of cell-bound gp120, sICs (left panel), or surface CD4 expression (right panel) on gp120_IIIB_-pulsed or untreated qCD4s. The gp120_IIIB_-pulsed qCD4s were further incubated with HIV-1^+^ Pt serum (Pt-serum) to form sICs or untreated and cultured in the absence or presence of anti-CD3 Abs. The effect of T22 pre-exposure on cell-bound gp120_IIIB_ in anti-CD3 Ab stimulation (αCD3+T22) was also examined. (**g**) Time course of cell-bound gp120, sICs, or surface CD4 expression on HIV-1_Lai_ (Lai)**-** (**left**), HIV-1_BaL_ (BaL)**-**, or clinical isolate (Clinical)**-** (**right**) pulsed qCD4s. HIV-1-pulsed qCD4s were further incubated with HIV-1^+^ Pt serum (**Pt-serum**) to form sICs or untreated and cultured in the absence or presence of anti-CD3 Abs. The effect of enfuvirtide (**Enf**) exposure was also examined. (**h**) The amount and location of cell-bound gp120 in gp120_IIIB_-pulsed qCD4s that were cultured in the absence or presence of anti-CD3 Abs were assessed by confocal microscopy (**upper**) or by western blotting (**bottom**). The lower numbers indicate the value by densitometry. (**i**) Time course of chemotaxis inhibition on gp120_IIIB_-, gp120_BaL_- (**upper**), or HIV-1_Lai_- (**bottom**) pulsed qCD4s. Chemotaxis of gp120- or HIV-1-pulsed or non-pulsed qCD4s toward the indicated chemokines was evaluated using a transwell assay. Bars, SD. The data here are representative of at least three independent experiments.

To investigate how the dynamics of the receptor are influenced by cellular state, we first examined the effect of cellular activation on VR surface expression. In agreement with previous studies [34]–[36], CXCR4 was rapidly internalized following anti-CD3 Ab-induced activation (**Fig. 1A** **left and 1B**). In contrast, CD4 expression remained virtually unaffected by anti-CD3 Ab treatment (**Fig. 1A** **right**). The addition of IL-2 or anti-CD28 Ab exposure along with anti-CD3 Ab treatment only had a marginal effect on initial CXCR4 internalization; however, these additional stimuli slightly enhanced the restoration of surface CXCR4 expression after 72 h (**Fig. 1A** **left**). In contrast, surface CD4 expression remained unaffected (**Fig. 1A** **right**). Collectively, we conclude that anti-CD3 Ab-triggered CD4^+^ T cell activation significantly alters CXCR4 dynamics but only has a marginal effect on CD4 dynamics.

We next evaluated VR turnover kinetics in qCD4s compared with lymphoma cells or activated cells. In these experiments, in addition to cycloheximide (a protein synthesis inhibitor), retrograde trafficking of internalized molecules and anterograde transport from the endoplasmic reticulum to the Golgi complex was blocked using Brefeldin-A (BFA) (see the schematic description of the inhibitors of protein turnover in **Fig. S1A**). Previous studies have shown that the effect of BFA on protein transport is greatest soon after treatment; therefore, the rate of reduction of VRs was determined after the first 2–3 h. CXCR4 expression was modestly reduced (approximately 25% in a 3-h assay) in T cell lymphoma A3.01 cells by BFA (**Fig. S1B left**). Some of the BFA-induced CXCR4 reduction (approximately 10% after 3 h) was caused by blockage of the transport of newly synthesized molecules, as shown using treatment with cycloheximide (**Fig. S1B left**). Because the transport of newly synthesized CXCR4 appeared to be suppressed by BFA, cycloheximide plus BFA did not produce any additive effects on CXCR4 reduction. Therefore, in agreement with a previous report [24], CXCR4 expression levels in A3.01 cells appear to be maintained by both recycling and replacement at relatively rapid rates. Next, we utilized qCD4s that were activated by 72 h of anti-CD3 Ab plus anti-CD28 Ab exposure but still had low CXCR4 expression (**Fig. 1A** **left**). In contrast with A3.01 cells, when we examined the pharmacological effects on VRs after 72 h of anti-CD3 Ab plus anti-CD28 Ab activation in qCD4s, CXCR4 expression was significantly reduced by both BFA (approximately 70% after 2.5 h) and cycloheximide treatments (approximately 60% after 2.5 h) (**Fig. S1B right; see also Fig. S1A**). Again, because the transport of newly synthesized CXCR4 appeared to be suppressed by BFA, cycloheximide plus BFA did not show any additive effects on CXCR4 reduction, suggesting that the reduced CXCR4 surface expression on activated qCD4s after 72 h of exposure was linked to rapid turnover due to greater degradation of CXCR4 than replacement by both newly synthesized and recycled molecules. In agreement with these results, RT-PCR analysis revealed that CXCR4 mRNA transcripts increased approximately 3.5-fold in activated qCD4s relative to qCD4s (**data not shown**). Utilizing confocal microscopy, we found that a significant portion of intracellular CXCR4 colocalized with the late endosomal/lysosomal marker LAMP-1 and the early endosomal marker Rab5 [37] in activated qCD4s, whereas, such colocalization was not observed in qCD4s (**data not shown**), suggesting the degradation of the CXCR4 proteins that are enhanced in activated qCD4s. Collectively, these results suggest that the CXCR4 turnover rate was enhanced because protein degradation predominated over replacement by both newly synthesized and recycled molecules; consequently, CXCR4 expression remains low in activated qCD4s.

In contrast, exposure of qCD4s to BFA minimally reduced CXCR4 expression levels following 16 h of incubation (approximately 3% and 20% after 2.5 h and 16 h, respectively), and exposure to both cycloheximide and Actinomycin-D (ActD), a DNA transcription suppressor, did not affect CXCR4 expression levels (**Fig. 1C** **left**). Again, cycloheximide plus BFA did not show any additive effects on CXCR4 expression. These results suggest that CXCR4 expression in qCD4s is stable and that a small fraction (approximately 3% over 3 h) of surface CXCR4 is continually internalized and recycled back to the surface. In contrast, CD4 expression in qCD4s was unaffected by exposure to BFA, cycloheximide, and ActD after 24 h (**Fig. 1C** **right**), indicating that CD4 turnover in qCD4s is more stable than CXCR4. Given that the inhibitors’ effects on protein transport/synthesis are not complete, it seems reasonable to propose that the actual turnover rate of VRs may be faster.

To further confirm the CXCR4 turnover results described above, we monitored CXCR4 turnover by employing T22, a peptide that binds to CXCR4 and blocks the binding of anti-CXCR4 mAb 12G5 [38]. The binding of 12G5 to CXCR4 was initially completely blocked by T22 exposure but gradually recovered (**Fig. S1C**). The duration and level of T22 occupancy on CXCR4 molecules over time is mainly influenced by four factors, namely CXCR4 internalization and degradation, CXCR4 recycling, *de novo* CXCR4 synthesis, and a steady level of T22 detachment from CXCR4; however, of these four factors, the rate of T22 detachment from CXCR4 is less likely to be affected by the level of CXCR4 turnover. Therefore, the duration of T22-CXCR4 occupancy should represent the level of intracellular replacement and degradation of CXCR4. The calculated times required for T22 occupation to fall to 50% of CXCR4 molecules were approximately 30 h, 8 h, and 8.5 h in qCD4s, activated qCD4s, and A3.01 cells, respectively (**Fig. S1C**). Although there are concerns that partial inhibition and/or cytotoxicity of the inhibitors may interfere with an accurate determination of VR dynamics, an estimation performed with either inhibitors or T22 showed similar trends in A3.01 cells, qCD4s, and activated qCD4s. Therefore, we conclude that CXCR4 turnover in qCD4s is truly stable, with a small fraction of CXCR4 slowly recycled, whereas CXCR4 turnover is significantly more rapid in both lymphoma and activated qCD4s. In this respect, it has been shown that a rigid layer of cortical actin exists in qCD4s [31], which may be partially linked to the slow turnover of VRs in qCD4s.

### Slow Turnover of Cell-bound HIV-1/gp120 on Dense Resting CD4^+^ T Cells

We next studied the dynamics of cell-bound gp120 in qCD4s before and after activation. Cells were exposed to the indicated subtypes of gp120 or HIV-1 for 30 min, thoroughly washed, and cultured at 37°C. As expected, confocal microscopy revealed that gp120, CD4, and CXCR4 colocalized on the surface in X4-gp120 (gp120_IIIB_)- or X4-HIV-1 (HIV-1_Lai_)-exposed qCD4s, whereas CXCR4 was not recruited to gp120-CD4 complexes on R5-gp120 (gp120_BaL_)-exposed qCD4s (**Fig. 1D**). The rates of surface gp120 reduction in both X4-gp120- and X4-HIV-1-exposed qCD4s were extremely slow, and we calculated that cell-bound gp120 was reduced to 50% at the surface approximately 3 d after exposure (**Fig. 1F** **left, open circles, 1G left, open circles, and Fig. S2A**). However, the rate of reduction of cell-bound X4-gp120 was rather rapid during the initial 20 h in both X4-gp120- and X4-HIV-1-exposed qCD4s (**Fig. 1F** **left, open circles and 1G left, open circles**). This observation may reflect that the rate of VR turnover is slightly enhanced by gp120 inducing VR-mediated signaling. In contrast, anti-CD3 Ab stimulation of X4-gp120- or X4-HIV-1-exposed qCD4s led to the rapid internalization of gp120-CD4-CXCR4 ternary complexes (see induction of gp120 internalization in **Fig. 1F** **left and G left**, open squares; see that CD4 and CXCR4 co-internalization only occurred in X4-gp120- or X4-HIV-1-exposed cells in **Fig. 1E**). Additionally, anti-CD3 Ab stimulation only induced CD4 down-regulation in X4-gp120- or X4-HIV-1-treated cells, which indicates that CD4 co-mobilizes with CXCR4 through gp120 (**Fig. 1E, 1F** **right** and **1G left**; see also **Fig. S2A**). However, T22 pre-exposure, which inhibits the association of gp120 with CXCR4, abrogated anti-CD3 Ab-induced gp120 internalization (**Fig. 1F**, closed triangles), suggesting that association with CXCR4 is essential for gp120 internalization. Therefore, the rapid internalization of gp120 in anti-CD3 Ab-stimulated qCD4s was mainly directed by internalized CXCR4.

When cell-bound gp120 stability was assessed by western blotting, approximately 70% of the gp120 was detected on the surface of qCD4s after 36 h of cell culture (**Fig. 1H** **, lower panel**; see also gp120 colocalizes with CD4 and CXCR4 after 36 h of cell culture in **Fig. 1H** **, upper panel**), and the results were comparable to those from FACS (**see** **Fig. 1F** **left, open circles**). In contrast, approximately 95% of the gp120 that was initially bound was degraded within 36 h of anti-CD3 Ab treatment (**Fig. 1H** **, lower panel; see also** **Fig. 1F** **left, open squares**). Because CCR5 expression was limited to approximately 10% of peripheral qCD4s (**see** **Fig. 1B**), most of the qCD4-bound R5-gp120 could bind to CD4 alone, and the dynamics of cell-bound R5-gp120 followed the dynamics of CD4. As anticipated, R5-HIV-1 (both the experimental strain (BaL) and the clinical isolate (Clinical)) on the qCD4 cell surface was retained for slightly longer than X4-HIV-1 (**Fig. 1G** **right, open circles and open diamonds**). Collectively, these results clearly demonstrate that irrespective of HIV-1 subtype, gp120 bound to qCD4s remains on the surface for a long time.

Because gp120 can be rapidly dissociated from virions by soluble CD4 [39], we hypothesized that gp120 dissociates from virions after HIV-1 becomes attached to surface CD4 and persists on VRs. To investigate this possibility, we used enfuvirtide (Enf) to inhibit virus and target membrane fusion [40], and we examined the dynamics of gp120 and p24, an HIV-1 capsid antigen, in HIV-1-exposed qCD4s. The dynamics of surface gp120 within 24 h of HIV-1 exposure in qCD4s were comparable between Enf-treated and untreated cells (**Fig. 1G** **left**, compare open circles with open diamonds). Similarly, western blotting analysis revealed that approximately 70% of the initially attached p24 disappeared from both Enf-treated and untreated cells after 24 h (**Fig. S2B**). However, early HIV-1 DNA products were only detected in untreated qCD4s **(data not shown)**. Therefore, irrespective of HIV-1 cell entry or uncoating, binding of HIV-1 to VRs appears to lead to the dissociation of gp120 from HIV-1, and the dissociated gp120 remains on VRs.

We then inquired whether gp120 directly associates with VRs for a prolonged period or associates with another molecule on the cell surface when cells are exposed to gp120 or HIV-1. Because the spatial resolution of confocal microscopy is not sufficient to determine gp120 directly associates with VRs accurately, we employed a transwell chemotaxis assay to examine the effect of X4-HIV-1 or gp120 exposure on CXCL12-induced chemotaxis. Given that X4-gp120 blocks the binding of CXCL12 to CXCR4, the initial exposure of qCD4s to X4-gp120 or X4-HIV-1 abrogated CXCL12-induced chemotaxis **(** **Fig. 1I** **, see open triangles (upper panel) and open squares (lower panel))**. However, CXCL12-induced chemotaxis was not suppressed by R5-gp120 exposure (**Fig. 1I** **upper panel, see open squares**). Consistent with the kinetics of cell-bound X4-gp120 or X4-HIV-1 in qCD4s, inhibition of CXCL12-induced chemotaxis was sustained for more than 3 d. In contrast, the migration of qCD4s toward CCL19 was not abrogated in X4-gp120- or X4-HIV-1-exposed qCD4s, serving as a control for the functional integrity of the cells to respond to other chemokines. These results cannot rule out the possibility that gp120 binds to other cell surface molecules but do clearly show that cell-bound X4-gp120 or X4-HIV-1 forms gp120-CD4-CXCR4 ternary surface complexes on qCD4s for prolonged periods.

### Slow Turnover of Ig-gp120 sICs on Dense Resting CD4^+^ T Cells

Several studies have shown that in the presence of serum from HIV-1^+^ Pts, sICs can form on HIV-1-infected cells or gp120-exposed uninfected cells (e.g., [15]). We tested whether patient serum contains sufficient anti-env Abs to allow the formation of sICs on gp120-pre-exposed qCD4s. Although the amount of sICs on qCD4s was proportional to the concentration of exposed gp120, the levels of sICs varied among patients, reflecting different levels of anti-env Abs in the serum of HIV-1^+^ Pts **(Fig. S3 right panel**; see also the **left panel**, which demonstrates the relationship between the concentration of exposed gp120 and the level of CD4 occupancy by gp120 by utilizing the gp120-blocking anti-CD4 mAb Leu3a and the gp120-non-blocking anti-CD4 mAb CD4V4**)**. We then examined the turnover of cell-bound gp120. The turnover of cell-bound gp120 was not significantly affected, even in the presence of patient serum **(compare open circles vs. open triangles (** **Fig. 1F and 1G** **) or open diamonds vs. open squares (** **Fig. 1G** **right))**. Collectively, serum from HIV-1^+^ Pts always contained sufficient levels of anti-env Abs to form sICs, and the kinetics of surface gp120 were extremely slow in qCD4s regardless of whether cell-bound gp120 formed sICs.

### Resting CD4^+^ T Cells from Acutely and Chronically HIV-1-infected Subjects are Coated with IgG and IgM

If gp120 turnover on qCD4s *in vivo* is similar to that observed *in vitro*, we should detect sICs on qCD4s from the peripheral blood of HIV-1^+^ Pts. For technical convenience, to easily detect qCD4s by FACS, we examined the presence of sICs in peripheral blood CD25^−^ CD69^−^ CD4^+^ CD3^+^ cells (designated here as resting CD4^+^ T cells; rCD4s). We utilized biotinylated anti-IgG F(ab′)_2_ and/or anti-IgM F(ab′)_2_ Abs to prevent non-specific surface binding through the Fc portion. Sixteen individuals with asymptomatic chronic HIV-1 infection, four individuals with acute HIV-1 infection, and ten healthy individuals were examined.

**Figure 2:**
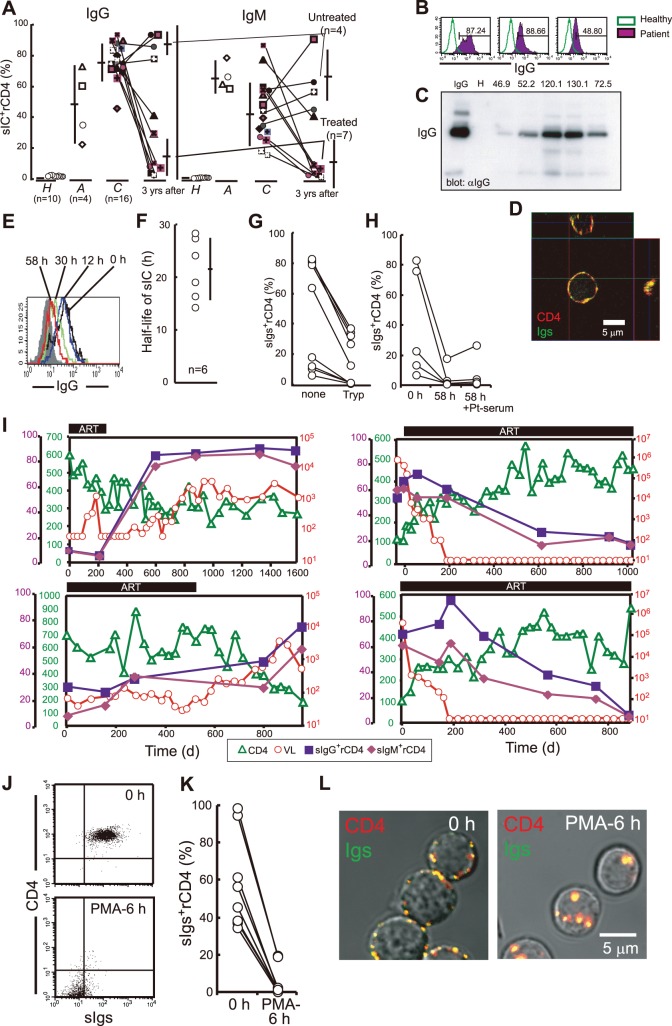
sICs of IgG or IgM on purified rCD4s from HIV-1^+^ Pts is molecularly linked to surface CD4 and shows slow turnover. (a) Summary of the percentages of IgG^+^ rCD4s or IgM^+^ rCD4s in healthy individuals (*H*), acute HIV-1^+^ individuals (*A*), and chronic pre-symptomatic HIV-1^+^ individuals (*C*) before, after 3 yrs of complete suppression of VL (<50 copies/ml) with ART (Treated), or untreated for 3 yrs (Untreated). Bars, SD. (b) Representative FACS of IgG expression on rCD4s from HIV-1^+^ Pts. (c) Anti-IgG Ab immunoblotting of purified HIV-1^+^ Pt rCD4 lysates. For the comparison of IgG binding levels, MFI values of IgG on rCD4s of the lysate samples are denoted above. IgG, positive IgG control; H, rCD4 lysate from an HIV-1-seronegative healthy donor. (d) Three-dimensional reconstitution confocal micrographs of Igs (Qdot655, green) and CD4 (Cy2, red) in rCD4s from an HIV-1^+^ Pt. (e, f) Representative time course of FACS (e) and calculated half-life of sICs (f) in purified rCD4s from an HIV-1^+^ Pt. Bar, SD. (g) Percentage of Ig^+^ cells in purified HIV-1^+^ Pt rCD4s without (none) or with 10 min of 0.05% trypsinization (Tryp). (h) Percentage of Ig^+^ cells in purified HIV-1^+^ Pt rCD4s before (0 h), after 58 h of culture (58 h), or 58 h of culture with exposure to HIV-1^+^ Pt serum (58 h+Pt-serum). (i) Changes in percentages of IgM^+^ or IgG^+^ rCD4s in blood, plasma VL, and CD4 lymphocyte counts during ART in the four HIV-1^+^ Pts. Two patients discontinued therapy after substantial suppression of VLs (left panels). HIV-1 RNA levels in two other patients were suppressed to undetectable levels for approximately 2 yr with ART (right panels). (j, k) Summary of the percentages (k) and representative FACS (j) of Igs on purified HIV-1^+^ Pt rCD4s before and after 6 h of PMA (0.3 ng/ml) exposure. (l) Fluorescence and DIC images of purified HIV-1^+^ Pt rCD4s that were stained with anti-Ig Abs (Cy2, green) and goat polyclonal anti-CD4 (Cy3, red) before and after 6 h of PMA exposure. Data in d and l are representative of five independent experiments.

In agreement with previous studies[26]–[28], means of 78.18±11.77% (± SD) and 42.18±19.73% (± SD) of peripheral blood rCD4s from sixteen chronic HIV-1^+^ Pts stained positive with anti-IgG and anti-IgM, respectively, whereas no sIC^+^ rCD4s were detected in healthy donors (**Fig. 2A and B**). In contrast, means of 48.22±22.69% (± SD) and 72.10±9.20% (± SD) of peripheral blood rCD4s from four acute HIV-1^+^ Pts were positive for anti-IgG and anti-IgM, respectively **(** **Fig. 2A** **)**. To more clearly demonstrate that peripheral blood rCD4s from HIV-1^+^ Pts were coated with Ig, rCD4s were purified from HIV-1^+^ Pts, lysed, and immunoblotted with anti-IgG Ab. As shown in **Fig. 2C**, IgG was only detected in rCD4 lysates from HIV-1^+^ Pts but not from healthy donors. The level of IgG detected by immunoblotting correlated with the mean fluorescence intensities (MFIs) of surface IgG on rCD4s as detected by FACS **(** **Fig. 2C**, the numbers above the panel indicate the MFIs of IgG in rCD4s**)**. Furthermore, utilizing confocal microscopy, we found that Igs colocalized with surface CD4 on rCD4s purified from HIV-1^+^ Pts **(** **Fig. 2D**, see three-dimensional reconstruction confocal micrograph**)**. These results collectively confirm that Igs are attached to CD4 on peripheral blood rCD4s in HIV-1^+^ Pts.

### cICs in the Serum of Viremic HIV-1^+^ Pts are Sufficient to form sICs on B Cells but not Resting CD4^+^ T Cells

It has been reported that B cells and T cells from HIV-1^+^ Pts are covered with complement-opsonized cICs [41] or auto-Abs [27]. Therefore, we tested whether serum from HIV-1^+^ Pts contains sufficient levels of cICs or auto-Abs to form sICs on rCD4s. Before proceeding with the experiments, we first sought to determine whether complement receptors or the Fc receptor were expressed in B cells and rCD4s. As shown in **Table S1**, complement receptors (CRs) 1, 2, and 3 and FcγRII were expressed on B cells but not rCD4s from both healthy donors and HIV-1^+^ Pts; these findings suggest that cICs with or without complement opsonization could theoretically bind to B cells through the Fc region of IgG to Fc receptors and/or complement opsonization to CRs but not to rCD4s.

Because B cells intrinsically express IgG and/or IgM on the cell surface, it is difficult to directly detect surface cICs using anti-IgG or anti-IgM Abs without interfering with their expression. Therefore, to clearly identify cell-bound cICs, we utilized a purified CD20^+^ IgG^dull^ IgM^dull^ population from the blood of healthy donors (see **Fig. S4B, upper panel**). When this purified subset of B cells (CD20^+^ IgG^dull^ IgM^dull^) was incubated with the patients’ serum, the percentages of B cells coated with cICs as detected by anti-IgG or anti-IgM Abs approximately paralleled viral loads (VLs) in the serum samples (**Fig. S4A and B**). In contrast, no cIC binding was detected on B cells incubated with serum from healthy donors or aviremic HIV-1^+^ Pts. Similarly, an *in situ* hybridization assay demonstrated that HIV-1 RNA was detected on all B cells incubated with serum from viremic HIV-1^+^ Pts but not from healthy donors or aviremic HIV-1^+^ Pts (**Fig. S4C and D**). In contrast, when rCD4s were incubated with serum from HIV-1^+^ Pts, virtually no cICs bound to the rCD4 cell surface (**Fig. S4E left column**). However, when gp120 pre-exposed cells were utilized, sICs were easily detected on all rCD4s incubated with serum from HIV-1^+^ Pts but not from healthy donors (**Fig. S4E middle column**). When rCD4s were exposed to 10 mg/ml of purified IgG from serum from HIV-1^+^ Pts, no Ig was detected on the rCD4s **(Fig. S4E right column)**. These results suggest that CR or FcγRII expression is critical for efficient cIC binding to the cell surface. Collectively, we can conclude that cIC levels in serum from viremic HIV-1^+^ Pts are insufficient to form sICs on rCD4s and that auto-Abs to rCD4s are either non-existent or below the limit of detection in serum from HIV-1^+^ Pts.

### The Dynamics of sICs on Resting CD4^+^ T Cells from HIV-1-infected Subjects show Similar Kinetics to gp120-Igs

We attempted to clarify whether sIC^+^ rCD4s in the peripheral blood of HIV^+^ Pts were also caused by cell-bound gp120. To this end, we first studied the dynamics of sICs in rCD4s purified from HIV^+^ Pts and sought to determine whether the dynamics are similar to Ig-gp120-VRs. The estimated mean duration of a 50% reduction in sICs in purified peripheral rCD4s from six patients was 21.76±5.62 h (± SD) (**Fig. 2E and F**). rCD4s should contain a certain number of cells in stages beyond G_0_. Therefore, the turnover of VRs and/or sICs in purified rCD4s may be much faster than in qCD4s; taking this into account, the calculated half-life of sICs on the patients’ rCD4s roughly matched the turnover of sICs in qCD4s. More importantly, trypsin treatment to remove trypsin-sensitive cell surface molecules (e.g., CD4) significantly reduced the level of sICs **(** **Fig. 2G** **)**. Similarly, once sIC levels were reduced in rCD4s, the levels were not restored by exposing the cells to Pt serum **(** **Fig. 2H** **)**, suggesting that rCD4s from HIV-1^+^ Pts either do not allow attachment of cICs or do not express surface epitopes for auto-Abs in Pt serum. Collectively, sICs on the patients’ rCD4s consisted of cell-bound molecules with similar kinetics to gp120-Igs.

### Longitudinal Cohort Analysis Reveals that Cell-bound HIV-1 or Related Molecules are Involved in the Formation of sICs on Resting CD4^+^ T Cells in Vivo

To further characterize whether sICs on patients’ rCD4s are linked to cell-bound HIV-1 molecules, we examined the levels of sIC^+^ rCD4s in the peripheral blood of antiretroviral therapy (ART)-experienced HIV-1^+^ Pts with longitudinal follow-up samples. Eleven individuals with asymptomatic chronic HIV-1 infection were examined. All 11 Pts with asymptomatic chronic HIV-1 infection were followed on an outpatient basis for >3 years (yrs) and were either treated with ART to complete suppression (<50 RNA copies/ml) or untreated. Means of 78.53±7.37% and 43.89±21.73% (± SD) of rCD4s in blood from 11 chronic HIV-1^+^ Pts stained positive with anti-IgG and anti-IgM Abs, respectively. However, in the 7 subjects for whom treatment led to complete suppression (<50 RNA copies/ml) of plasma VL for 3 yrs, the percentages of sIC^+^ rCD4s were significantly reduced, with means of 15.28±13.36% (± SD) (vs. 79.87±6.46% (± SD, = before treatment), P<0.0001) and 4.71±2.49% (± SD) (vs. 46.25±29.29% (± SD, = before treatment), P = 0.0045) of rCD4s in blood positive for IgG and IgM, respectively. In contrast, in the four HIV-1^+^ Pts who remained untreated for 3 yrs, the number of sIC^+^ rCD4s in blood significantly increased, with means of 89.75±8.53% (± SD) (vs. 73.75±9.03% (± SD, before), P = 0.036) and 63.21±16.18% (± SD) (vs. 42.75±13.45% (± SD, before), P = 0.0091) of rCD4s positive for IgG and IgM, respectively.

In **Fig. 2I**, four representative chronic HIV-1^+^ Pts who had frequent peripheral blood sampling for CD4 or viral RNA testing are shown. After initiating ART, plasma virus became undetectable (<50 RNA copies/ml) within 200 days in two subjects (**right panels**). In these two subjects, both IgG^+^ and IgM^+^ rCD4s gradually decreased in the peripheral blood; however, it required approximately 2 yrs for the percentages of IgG^+^ and IgM^+^ rCD4s to reach less than approximately 10%. In contrast, in the two subjects with treatment interruption, the percentages of both IgG^+^ and IgM^+^ rCD4s promptly increased (**left panels**). Although the change in frequency of IgG^+^ and IgM^+^ rCD4s in blood was relatively slow compared with the change in plasma VLs, the frequencies in peripheral blood correlated to plasma VLs **(** **Fig. 2I** **)**. Therefore, these results collectively indicate that at least cell-bound HIV-1 or related molecules are involved in the formation of sICs on rCD4s *in vivo*. Interestingly, the percentage of both IgG^+^ and IgM^+^ rCD4s appears to be inversely correlated to the number of CD4^+^ T cells in peripheral blood (**Fig. 2I**, compare closed squares or closed diamonds with open triangles).

### sICs are Attached to Surface CD4 on Resting CD4^+^ T Cells from HIV-1-infected Subjects

Next, we investigated whether colocalized sICs and CD4 were molecularly linked. To examine this possibility, rCD4s purified from HIV-1^+^ Pts were exposed to phorbol myristate acetate (PMA) to induce CD4 internalization and determine whether sICs could co-mobilize with CD4. After 6 h of PMA stimulation, CD4 and most of the sICs had disappeared from the cell surface as determined by FACS (**Fig. 2J and K**). Confocal microscopy revealed that sICs colocalized with surface CD4 were rapidly co-internalized into the cells after 6 h of PMA stimulation (**Fig. 2L**). Collectively, sICs were molecularly linked to surface CD4 on rCD4s purified from HIV-1^+^ Pts.

### The gp120-binding Domains of Surface CD4 are Occupied on Resting CD4^+^ T Cells from HIV-1-infected Subjects

**Figure 3:**
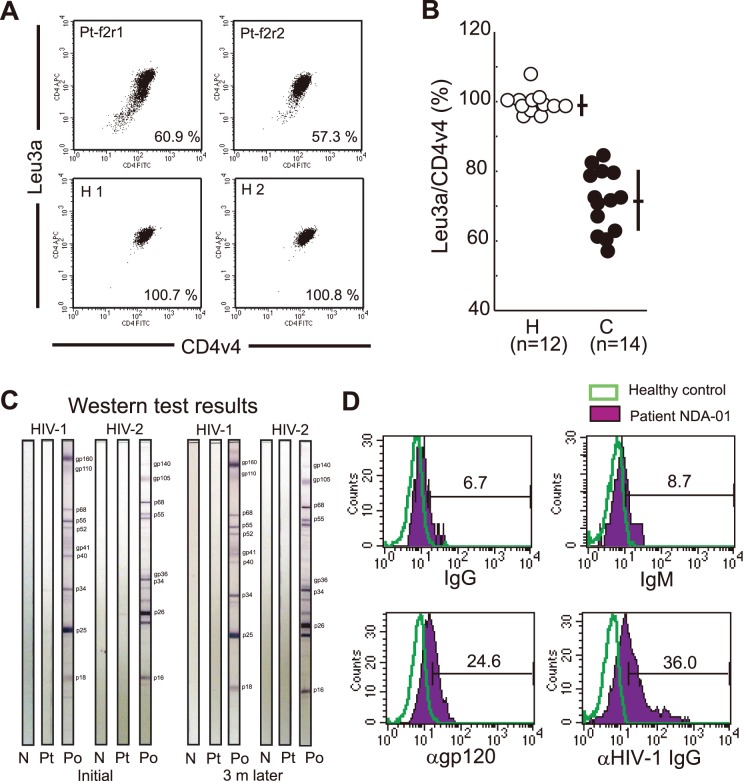
rCD4s from HIV-1^+^ Pts are coated with gp120. (**a**) Representative FACS data from rCD4s purified from healthy controls (H1, H2) or chronic asymptomatic patients (Pt-f2r1, Pt-f2r2) stained with Leu3a and CD4v4 (numbers in FACS plots indicate percentages of MFIs of Leu3a/CD4v4). (**b**) Summary of results of percentages of MFIs of Leu3a/CD4v4 in purified rCD4s from healthy controls (H) and chronic asymptomatic patients (C; CD4 counts: 420±84.6 (± SD); IgG^+^ rCD4s: 75.5±12.6% (± SD)). (**c, d**) Detection of cell-bound gp120 on rCD4s in a patient with low anti-gp120 Ab levels. (**c**) Western blot test results for the HIV-1^+^ Pt (NDA-01) at initial admission and three months after. N, negative control; Pt, patient serum; Po, positive control. HIV-1 infection was defined as detectable amounts of plasma HIV-1 RNA (1.5×10^5^ copies/ml at initial admission), a positive antibody test (HIV1/2 ELISA), and low CD4^+^ T cell counts (38 cells/µl). Plasma HIV-1 env and gag region sequences revealed that the patient was infected with a clade B HIV-1. (**d**) FACS data from rCD4s stained with anti-IgG (upper left), anti-IgM (upper right), anti-gp120 (rabbit anti-gp120 antiserum) (lower left), or purified IgG from pooled serum from HIV-1^+^ Pts (lower right).

To further confirm whether gp120 was actually bound to CD4 on the patients’ rCD4s, we employed two Abs, namely, gp120-blocking anti-CD4 mAb Leu3a and the mAb CD4-v4, which does not block the binding of gp120 to CD4. When we compared the MFIs of Leu3a with those of CD4-v4, the MFIs of Leu3a were always significantly lower than those of CD4-v4 in peripheral rCD4s from HIV-1^+^ Pts but not from healthy controls. This finding suggests that the gp120-binding domains of surface CD4 molecules were occupied in rCD4s from HIV-1^+^ Pts **(** **Fig. 3A and 3B**, percentages of Leu3a/CD4v4 from healthy donors and HIV-1^+^ Pts are 100.1±3.51% and 70.61±9.09% (± SD), respectively; P<0.0001; see also in **Fig. S3 left** for the correlation between the concentration of gp120 exposed to qCD4s and the degree of blocking from Leu3a binding to CD4**)**.

### Direct gp120 Detection on Resting CD4^+^ T Cells from an HIV-1-seronegative Chronically HIV-1-infected Subject

We had the opportunity to directly examine cell-bound gp120 in rCD4s purified from a patient whose anti-env Ab levels were below the limit of detection of a conventional clinical western blotting test **(** **Fig. 3C**, see the results of the western blotting test at initial admission and 3 months later**)**. We assumed that if gp120 were attached to rCD4s *in vivo*, the attached gp120 would not be or be only weakly coated with anti-env Abs in such a Pt. In other words, the epitopes for anti-env Abs would only be loosely occupied. Therefore, if such rCD4s were directly stained with anti-env Abs or purified IgG *in vitro*, we could directly detect gp120 on the cell surface. As expected, the rCD4s were only weakly positive for sICs (**Fig. 3D** **, upper panels**). However, when stained with an anti-env Ab or a mixture of purified IgG from HIV-1^+^ Pts, a significant portion of the rCD4s stained positive (**Fig. 3D** **, lower panels**). Therefore, attachment of gp120 to the surface of rCD4s was demonstrated directly in a patient whose anti-env Ab levels were below the limit of detection. Collectively, these results clearly demonstrate that sICs on rCD4s in HIV^+^ Pts link to cell-bound gp120.

### sIC^+^ Resting CD4^+^ T Cells Activate Phagocytosis by Macrophages

**Figure 4:**
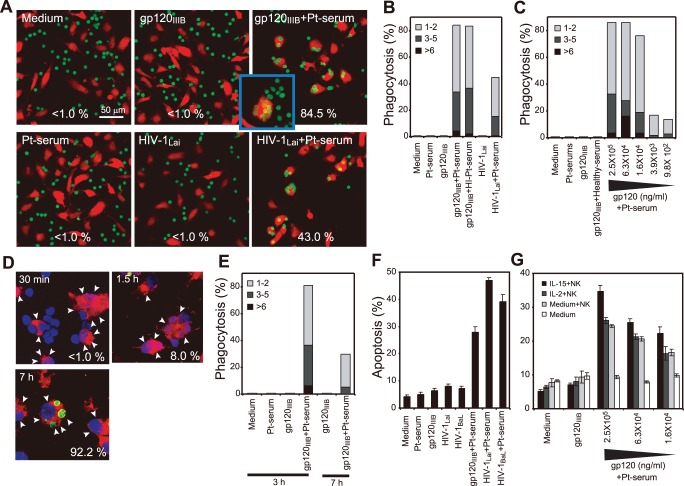
sICs on qCD4s trigger Fc-mediated effector systems. (**a–e**) Autologous macrophages phagocytose qCD4s with sICs. Orange-CMTMR-labeled macrophages (red) cocultured with CFSE-labeled autologous qCD4s (green) exposed to the indicated concentrations of gp120_IIIB_, HIV-1_Lai_, or medium followed by incubation with HI patient serum (Pt-serum), non-HI patient serum, or medium for 1 h before coculture. (**a–c**) Confocal micrographs of representative data (**a**) and summary of phagocytosis assays (**b, c**) shown as percentages of macrophages containing 1–2, 3–5, or >6 qCD4s. The numbers in (**a**) denote percentages of macrophages containing at least one qCD4; inset shows a macrophage containing >10 qCD4s. (**d**) Time course of TUNEL assay on sIC^+^ qCD4s that were phagocytosed by macrophages. Confocal images of macrophages (arrowheads, red), TUNEL^+^ (green) and cell nuclei (Topro-3, blue). The numbers indicate the percentage of TUNEL^+^ phagocytosed qCD4s/total phagocytosed qCD4s. (**e**) Summary of the time course of phagocytosis assays. (**f**) Summary of apoptotic qCD4s in the NK cell-mediated ADCC assay. CFSE-labeled NK cells incubated with autologous qCD4s (2∶1), which were exposed to the indicated concentrations of gp120_IIIB_, gp120_BaL_, HIV-1_Lai_, HIV-1_BaL_, or medium. (G) Summary of effects of IL-2 (50 ng/ml) or IL-15 (20 ng/ml) treatment on NK cell-mediated ADCC. Bars, SD. The data presented here are representative of at least three independent experiments.

We next investigated the pathological role of sICs on rCD4s. To this end, we examined whether sICs bound to rCD4s could trigger Fc-mediated effector systems. We first examined whether sICs that formed *in vitro* on HIV-1/gp120-pre-exposed qCD4s could trigger ADCP by autologous macrophages **(** **Fig. 4A–E** **).** As expected, qCD4s exposed to medium, HIV-1^+^ Pt serum, or HIV-1 or gp120 alone did not trigger phagocytosis by macrophages **(** **Fig. 4A** **, left and middle panels,** **Fig. 4B** **, Fig. S5A, and Movie S1)**. In contrast, sICs that formed *in vitro* on HIV-1- or gp120-pre-exposed qCD4s triggered 84.5% and 43.0% of the macrophages to phagocytose more than one qCD4, respectively **(** **Fig. 4A, right panels**, **Fig. 4B** **, Fig. S5B, Fig. S6, and Movie S2)**. The percentage of macrophages that phagocytosed qCD4s increased in proportion to the MFIs of sICs on qCD4s **(** **Fig. 4C** **)**. In contrast, regardless of the usage of heat-inactivated (HI) serum or non-HI serum to form sICs on gp120-pre-exposed qCD4s, there was no difference in the levels of macrophage phagocytosis (**Fig. 4B**). Because heat inactivation eliminates the function of complement, phagocytosis of sIC^+^ qCD4s should be predominantly induced through Fc-mediated pathways. Our time course study and live cell imaging of phagocytosis revealed that the attachment and engulfment of sIC^+^ qCD4s by macrophages started immediately after coculture began, and phagocytosis of sIC^+^ qCD4s finished within 1.5 to 3 h (**Fig. 4D, E** **, and Fig. S5B**). As shown using TUNEL staining, apoptosis of sIC^+^ qCD4s became noticeable only after phagocytosis was completed **(** **Fig. 4D** **)**. Therefore, the formation of sIC on gp120-exposed drCD4 was not sufficient for inducing cell death, and the induction of phagocytosis of sIC^+^ qCD4s was not related to apoptotic changes in the plasma membrane. After 7 h of coculture, apoptosis had occurred in 92% of the ingested qCD4s, and the apoptotic cells were rapidly digested (**Fig. 4E**, note that the percentage of macrophages containing qCD4s decreased from 82% to 32%).

We next examined whether sICs formed *in vitro* on HIV-1/gp120-pre-exposed qCD4s could trigger ADCC by autologous NK cells. As reported previously [1], [15]–[18], significant cell death was observed when sICs formed *in vitro* on HIV-1- or gp120-pre-exposed qCD4s were cocultured with NK cells in the presence of HIV-1^+^ Pt serum (**Fig. 4F and G**). The number of apoptotic qCD4s increased in proportion to the MFIs of sICs on qCD4s (**Fig. 4G**). Moreover, as reported previously [42]–[44], the ADCC response mediated by NK cells was enhanced by IL-2 or IL-15 exposure (**Fig. 4G**).

**Figure 5:**
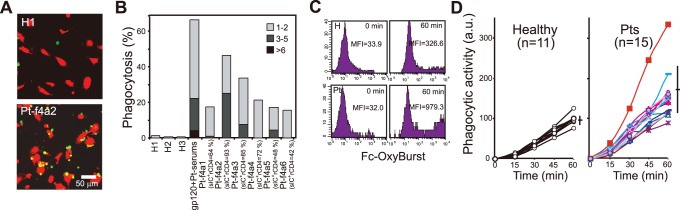
Patients’ sIC^+^ rCD4s trigger Fc-mediated effector systems. (**a, b**) Representative confocal micrographs (**a**) and a summary of phagocytosis assays (**b**) of purified rCD4s from healthy donors (H) or patients (Pt) with allogeneic macrophages from a healthy donor. Arrowheads indicate the macrophages containing qCD4s. (**c, d**) Representative FACS data (**c**) and summary of Fc-OxyBurst assays (**d**). Macrophages from healthy donors (Healthy) or chronic asymptomatic donors (Pts) were incubated with Fc-OxyBurst immune complexes (Molecular Probes) (10 mg/ml). The relative quantities of superoxide generated by macrophages were measured every 15 min using FACS. Bars, SD.

We then investigated whether purified rCD4s from HIV-1^+^ Pts could trigger Fc-mediated effector systems [45]. Allogeneic macrophages did not phagocytose rCD4s from healthy control subjects; however, a significant number of allogeneic macrophages phagocytosed rCD4s from more than one HIV-1^+^ Pt (**Fig. 5A and B**). Therefore, sIC^+^ rCD4s were sufficient to trigger an ADCP reaction to autologous macrophages.

Finally, we sought to determine whether monocytes from HIV-1^+^ Pts maintained the capacity to induce Fc-mediated phagocytosis. The phagocytic activities of freshly isolated macrophages from chronic HIV-1^+^ and healthy control subjects were measured directly using Fc-OxyBurst assays [46]. As shown in **Fig. 5C and D**, the phagocytic activities of freshly isolated macrophages from chronic, asymptomatic HIV-1^+^ Pts were significantly higher (151.25±56.19 vs. 99.25±14.2 (± SD), p<0.001) than those from the controls. These results collectively suggest that sIC^+^ rCD4s *in vivo* may be destroyed and removed by macrophages or NK cells through ADCP or ADCC, respectively.

### Frequencies and Numbers of sIC^+^ Resting CD4^+^ T Cells in Blood Increase after Spleen Removal

**Figure 6:**
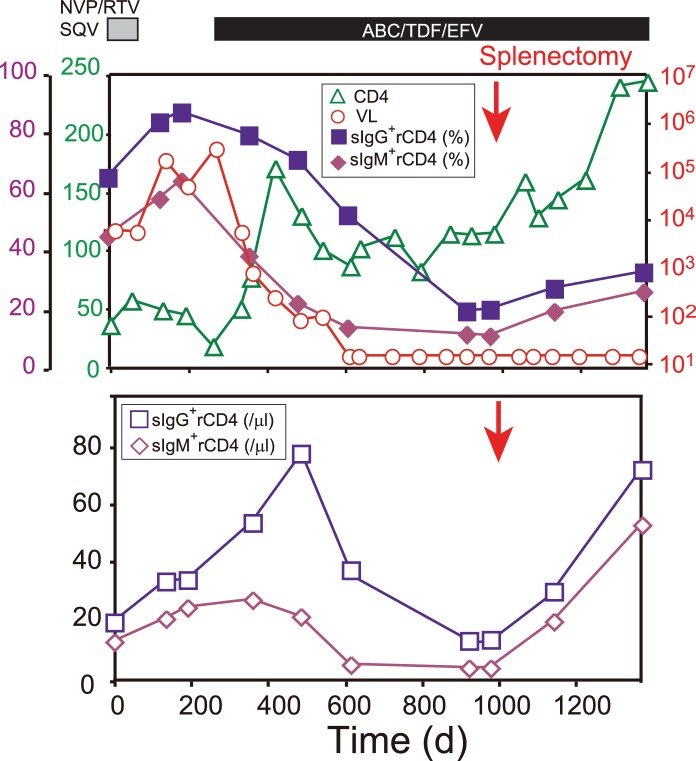
Frequencies and numbers of sIC^+^ rCD4s in the blood increase after spleen removal. Percentages of sIgG^+^ and sIgM^+^ rCD4s in the blood, plasma VL, and CD4 lymphocyte counts (upper), or absolute numbers of sIgG^+^ and sIgM^+^ rCD4s (lower) in a hemophiliac HIV-1^+^ Pt who had undergone splenectomy. Patient interrupted therapy with nevirapine, ritonavir, and saquinavir due to side effects and then initiated therapy with abacavir, tenofovir, and efavirenz. The arrow indicates the day on which the splenectomy was performed.

Finally, we performed a longitudinal analysis of sIC^+^ rCD4 levels in peripheral blood from an HIV-1-infected hemophiliac individual who underwent a splenectomy during the course of ART. When ART was discontinued due to side effects, the percentages and numbers of both IgG^+^ and IgM^+^ rCD4s rapidly increased in peripheral blood **(** **Fig. 6** **)** as shown in the previous section. Thereafter, ART with a different regime was initiated. Approximately 300 days after treatment, the plasma VL became undetectable (<50 RNA copies/ml), and the percentage and number of both IgG^+^ and IgM^+^ rCD4s gradually declined. However, the patient required a splenectomy for the treatment of a severe, uncontrolled epidural hemorrhage caused by immune thrombocytopenic purpura. Immediately after removal of the spleen, the percentages of IgG^+^ and IgM^+^ rCD4s increased to 11% and 22%, respectively, and the actual numbers of IgG^+^ and IgM^+^ rCD4s were markedly elevated from 15/µl and 5/µl to 82/µl and 55/µl, respectively, whereas VL remained undetectable. These results strongly suggest that substantial numbers of sIC^+^ rCD4s are trapped or eliminated from circulation in the spleen.

## Discussion

The presence of Ig^+^ CD4^+^ T cells in the blood of HIV-1^+^ Pts has been reported [26]–[28]; however, these studies examined the percentages of Ig^+^ CD4^+^ T cells utilizing FACS or related techniques alone. In this study, we first sought to determine whether peripheral blood rCD4s in HIV-1^+^ Pts are truly coated with IgG and/or IgM. We utilized biotinylated anti-IgG and/or anti-IgM F(ab′)_2_ Abs to prevent the non-specific surface binding of Abs through the Fc portion. Furthermore, we simultaneously measured Ig expression levels in rCD4s purified from HIV-1^+^ Pts or healthy individuals by FACS and immunoblotting. We confirmed that the levels of surface Ig on rCD4s detected by MFIs of anti-IgG by FACS approximately paralleled the levels of IgG detected by immunoblotting (**Fig. 2C**). Thus, we confirmed that peripheral rCD4s from HIV-1^+^ Pts are truly coated with Igs. In addition, utilizing confocal microscopy, we found that Igs co-localized with surface CD4 on rCD4s from HIV-1^+^ Pts (**Fig. 2D**) and co-mobilized with CD4 when inducing CD4 internalization by PMA exposure (**Fig. 2J–L**). Collectively, we demonstrated that Igs are attached to surface CD4 on peripheral rCD4s from HIV-1^+^ Pts.

A cohort study using peripheral blood samples showed that the percentages of Ig^+^ rCD4s from HIV-1^+^ Pts positively correlated with plasma VLs, suggesting that ICs were formed via HIV-1-related molecules on the cell surface (**Fig. 2A and I**). HIV-1 virions circulate in HIV-1^+^ Pt serum as cICs [47]. Some reports have suggested that peripheral Ig^+^ rCD4s may be linked to nonspecific attachment of cICs to the cell. Furthermore, the production of auto-Abs against peripheral rCD4s in HIV-1^+^ Pts has also been reported [27]. When B cells, which express both CRs and Fcγ receptors, were exposed to patient serum, sICs formed in an quantity that was relatively proportional to VL, whereas when qCD4s, which do not express CRs or Fcγ receptors, were exposed to patient serum, no sICs formed on the cells (**Fig. S4**). Additionally, we excluded the possibility that HIV-1^+^ Pt serum contains auto-Abs (**Fig. S4E left column**). Importantly, once Ig is lost from the surface of HIV-1^+^ Pt rCD4s after sustainable cell culture, no sICs formed on the surface even when exposed to patient serum (**Fig. 2H**), suggesting that rCD4s from HIV-1^+^ Pts do not express molecules that bind to cICs or self-antigens that react with certain Abs in Pt serum. Collectively, we have excluded the possibility that sICs are formed due to HIV-1^+^ Pt serum containing sufficient levels of auto-Abs or cICs.

Next, to clarify whether gp120 binds to CD4 molecules on rCD4s *in vivo* in HIV-1^+^ Pts, we showed that the gp120-binding domain of CD4 was occupied in rCD4s from HIV-1^+^ Pts (**Fig. 3A and B**), thereby indirectly demonstrating that gp120 is attached to CD4 on rCD4s *in vivo*. Furthermore, we showed that anti-env Abs directly bound to rCD4s from an HIV-1-infected individual whose anti-gp120 Ab levels were below the sensitivity of a conventional western blotting test (**Fig. 3C and D**). Collectively, we conclude that sICs on rCD4s in HIV-1^+^ Pts result from CD4-bound gp120. However, we can hypothesize that cell-bound gp120 could reflect the production of HIV-1 in rCD4s. In this respect, it is well established that direct infection of rCD4s does not lead to productive infection [**32]**, **[48]**, instead, resulting in a labile state known as preintegration latency. Therefore, gp120 attached to CD4 is not linked to HIV-1 production by rCD4s.

In contrast, *in vitro* culture of purified rCD4s from HIV-1^+^ Pts revealed that a 50% reduction in sICs on rCD4s required approximately 20 h (**Fig. 2E and F**) due to the slow turnover of VRs on rCD4s (**Fig. 1** **, Fig. S1 and S2**). Therefore, the half-life of sICs on rCD4s is much longer than the duration on CD4^+^ T cells recirculating between LNs and the peripheral blood [7]. Before sICs disappear from the surface, rCD4s may be continuously exposed to gp120 and/or HIV-1 at high concentrations in the lymphoid organs [5]. Thus, the levels and percentages of sIC^+^ rCD4s may become equilibrated to the levels of virus production and/or anti-HIV-1 Abs in the lymphoid organs.

To clarify the pathological effects of sICs on rCD4s, we demonstrated here that sIC^+^ rCD4s produced *in vitro* or isolated from HIV-1^+^ Pts ultimately induced ADCP and ADCC by autologous macrophages (**Fig. 4A–E** **,** **Fig. 5A and B** **, Fig. S5B, Fig. S6, and Movie S2**) and NK cells (**Fig. 4F and G**), respectively. Furthermore, the phagocytic activities of monocytes as measured directly using Fc-OxyBurst assays on freshly isolated monocytes from HIV-1^+^ Pts were even stronger in the healthy donors (**Fig. 5C and D**). Therefore, these results suggest that sIC^+^ rCD4s in peripheral blood may be destroyed and removed from circulation at a constant rate.

We found that the percentages of sIC^+^ rCD4s in HIV-1^+^ Pts were inversely correlated with the number of CD4^+^ T cells in the blood (**Fig. 2I**). Furthermore, we found that in an HIV-1-infected individual whose VL became undetectable with ART, the percentage and number of sIC^+^ rCD4s in blood gradually decreased but promptly increased after splenectomy (**Fig. 6**). Therefore, we can hypothesize that sIC^+^ rCD4s may be destroyed and removed from circulation by macrophages or NK cells in the spleen or other lymphoid organs. Indeed, splenomegaly is a common symptom of both acute and chronic HIV-1 infection [49]. Furthermore, we found that approximately 100% of patients’ IgM^+^ rCD4s were also coated with iC3b complement fragments, known as C3 opsonization (**data not shown**), suggesting that cell-bound IgM is capable of fixing complement and that IgM^+^ iC3b^+^ rCD4s may induce stronger ADCP activity by macrophages than IgG^+^ rCD4s.

CD4 molecules on CD4^+^ T cells play an important role in forming the immunological synapse between CD4^+^ T cells and antigen-presenting cells [50]. However, the attachment of ICs to CD4 molecules could interfere with normal immunological synapse formation between CD4^+^ T cells and antigen-presenting cells and suppress the full activation of CD4^+^ T cells. Therefore, our findings here can also be extended to explain the reduced immune function of CD4s in HIV-1^+^ Pts. However, future studies are needed to confirm this possibility.

**Figure 7:**
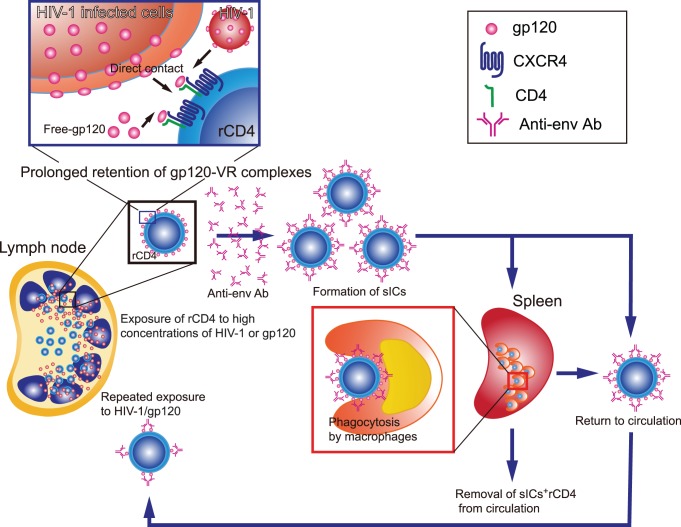
Schematic figure summarizes the causes and consequences of sIC^+^ rCD4s. rCD4s continuously travel between the blood stream and LNs over a period of approximately 1 d. Because a large proportion of HIV-1 is produced in the LNs, the target T cells that migrate to the LNs are exposed to high concentrations of HIV-1, gp120, or ICs as well as anti-env Abs. Prolonged retention of gp120-VR complexes on rCD4s causes the retention of sICs in a manner that reflects the levels of HIV-1 exposure in the LNs. sIC^+^ rCD4s are removed from circulation through ADCP or ADCC by macrophages or NK cells, respectively. The sIC^+^ rCD4s that are not removed from circulation remigrate to the LNs to be exposed to a high concentration of gp120/HIV-1. The percentages and amounts of sICs on rCD4s in the blood reflect a balance of five factors, namely, the levels of virus production in lymphoid tissues, the levels of anti-env Abs, the turnover dynamics of sICs on rCD4s, the duration of repeated exposure by continuous migration to the lymphoid organs, and the levels of immunological elimination of sIC^+^ rCD4s.

In **Fig. 7**, we summarized our hypothesis of the mechanisms of sIC formation on rCD4s and their effects on the dynamics of rCD4 circulation. In our model, the length of time that sICs remain on rCD4s was extremely long compared with CD4s that are circulating between the LNs; as a result, rCD4s continue to be exposed to high concentrations of HIV-1 in the lymphoid organs. Therefore, the percentages and levels of sICs on rCD4s equilibrate to HIV-1 production in the lymphoid organs. However, sIC^+^ rCD4s are also subject to immunological pressure from both macrophages and NK cells. Therefore, the percentages and levels of sICs on rCD4s were also at equilibrium with the degree of immunological pressure. Collectively, the percentages and levels of sICs on rCD4s in blood appear to reflect a complex interplay between the levels of virus production in lymphoid tissues, the levels of anti-env Abs, the rate of sIC removal from the cell surface, the duration of repeated exposures to HIV-1/gp120 or ICs, and the degree of immunological elimination and trapping of sIC^+^ rCD4s from peripheral circulation. Because ART may not dramatically influence sIC turnover rates on rCD4s, the levels of anti-env Abs, and the duration of CD4^+^ T cell circulation among lymphoid tissues, changes in the percentage of sIC^+^ rCD4s in the blood after initiation of ART may reflect the level of virus production in lymphoid tissues and the degree of immune pressure on sIC^+^ rCD4s. Importantly, our hypothesis here is highly consistent with the previously proposed mathematical model [9] that suggests that the effects of HIV-1 (e.g., induction of LN accumulation and cell death after entering the LNs) on resting T lymphocytes can explain the depletion of CD4^+^ T cells from the peripheral blood during HIV-1 infection.

When we analyzed the percentages of sIC^+^ rCD4s in blood, sIC^+^ rCD4s were detectable in peripheral blood after approximately 2 yrs of complete suppression of plasma VL (**Fig. 2A and I**). Effective ART has been shown to rapidly reduce the levels of both plasma VL and HIV-1-producing cells to undetectable levels; however, HIV-1 replication continues in LNs in patients with undetectable plasma VLs after ART [48], [51], [52]. If we assume that ART treatment does not significantly change the degree of immunological pressure on sIC^+^ rCD4s in our model, the number of sIC^+^ rCD4s should mainly reflect residual viral production in LNs. Therefore, monitoring the percentage of sIC^+^ rCD4s in peripheral blood may be a promising tool to examine residual virus replication in patients with undetectable plasma virus levels under ART.

More importantly, sIC^+^ rCD4s in blood were only found in HIV-1^+^ Pts; we did not find sIC^+^ rCD4s in healthy donors or any patients with other viral and bacterial infections and autoimmune diseases (**data not shown)**. Therefore, detection of sIC^+^ rCD4s in the blood in itself can be used as a marker to confirm the diagnosis of HIV-1 infection. Furthermore, our results suggest that monitoring the Ig subclasses of sICs or the percentage of sIC^+^ rCD4s may also be useful for determining the stage and progression of HIV-1 infection (**Fig. 2A**) because during acute infection, sICs largely consisted of IgM, and the percentage of sIC^+^ rCD4s gradually increased during the follow-up period (approximately 3 yrs) (**Fig. 2A**). Therefore, it is logical to use Ig^+^ rCD4s levels in blood as an independent clinical marker for easily confirming a diagnosis of HIV-1 infection, for identifying clinical stages, and for evaluating residual virus production under ART. Additionally, because sICs were mainly formed by anti-env Abs with non-neutralizing activity, the presence of sIC^+^ rCD4s may demonstrate that non-neutralizing anti-env Abs play a detrimental role in uninfected rCD4s. Therefore, preventing the induction of non-neutralizing anti-env Abs by vaccination may facilitate efficient immune responses against HIV-1 infection.

The results of the experiments we describe here were obtained using a limited number of clinical samples. Therefore, it is essential to perform detailed studies in the future utilizing a larger number of samples to answer the following questions: 1, Do sICs on rCD4s influence the dynamics of rCD4s *in vivo*? 2, Are sICs on rCD4s destroyed by ADCC and APCP *in vivo*? 3, As a result of affecting immunological synapse formation, do sICs on rCD4s induce anergy or suppress the full activation of rCD4s? 4, Can sICs on rCD4s be used for confirming the diagnosis of HIV-1 infection, for identifying the clinical stage, and for evaluating residual virus production under ART?

## Materials and Methods

### Reagents

Chemicals, Abs, and recombinant cytokines were purchased from Sigma (St. Louis, MO), BD (San Diego, CA), and R&D Systems (Minneapolis, MN), respectively, unless otherwise specified. Purified gp120_IIIB_ and recombinant gp120_BaL_ were obtained from Advanced Biotechnologies, Inc. (ABI, Columbia, MD) and the NIH AIDS Research and Reference Program, respectively. Aldritiol-2 (AT-2)-inactivated HIV-1_Lai_, HIV-1_BaL_, and clinical isolates were prepared as previously described [53], [54].

### Subjects and Research Ethics

PBMCs were collected from 49 ART-naive, HIV-1-infected individuals, 46 HIV-1-infected individuals undergoing ART and 54 HIV-1-seronegative individuals to study VR and surface Ig dynamics and to perform phagocytosis assays. The HIV-1^+^ individuals were classified as having acute or chronic asymptomatic infections. Acute infection was defined as having less than 14 d of symptoms of acute HIV-1 infection with the presence of HIV-1 RNA in the plasma and seroconversion by HIV-1/2-reactive western blots during follow-up. Chronic asymptomatic HIV-1 infection was defined as being seropositive for >1 yr with CD4 counts >250/µl without any symptoms of opportunistic infections. One HIV-1-seronegative healthy individual served as the negative control. Here, aviremic individuals were those with a plasma VL of <50 *HIV-1* RNA copies/ml. This study was approved (IMCJ-H14-60) by the National Center for Global Health and Medicine Ethical Committee, and written informed consent was obtained from every subject.

### Purification and Isolation of CD4^+^ T, B, and NK Cells

The highly purified drCD4s from healthy donors, which were used as representative qCD4 T cells *in vivo*, were purified by negative selection with magnetic beads followed by T cell density gradient separation on a discontinuous Percoll gradient (Pharmacia Biotech, Uppsala, Sweden) as described previously [34]. The rCD4s were purified using the CD4^+^ T Cell Isolation Kit II (Miltenyi Biotech, Auburn, CA) followed by magnetic depletion with anti-CD25 and anti-CD69 Abs. CD20^+^ IgG^dull^ IgM^dull^ B cells were isolated using the B Cell Isolation Kit II (Miltenyi Biotech) followed by magnetic depletion with anti-IgG and anti-IgM Abs. NK cells and macrophages were isolated using the NK Cell Isolation Kit II and the Macrophage Isolation Kit II (Miltenyi Biotech), respectively.

### Preparation of Activated qCD4s

Purified qCD4s were stimulated for 72 h with plate-bound anti-CD3 Abs (UCTH1∶40 mg/ml) and anti-CD28 Abs (Lew-28∶20 mg/ml) in RPMI 1640 containing fetal calf serum (FCS) at 37°C in humidified air containing 5% CO_2_.

### Kinetic Studies and Flow Cytometry

Kinetic studies of CXCR4 and CD4 expression were performed as previously described [30]. Briefly, A3.01 cells, qCD4s, and activated qCD4s were cultured in flat-bottom 96-well microtiter plates (Nalge Nunc, Penfield, NY) (in triplicate) with or without ActD (20 µg/ml), BFA (10 µg/ml), and/or cycloheximide (50 µg/ml). The concentrations of inhibitors used were as previously described [30]. The percent change in surface receptor expression was calculated from the MFIs (**except for** **Fig. 1A and B**) or percentages of cells in predetermined gates (**Fig. 1A and B**).

For kinetic studies, qCD4s were incubated on ice with gp120_IIIB_ (250 ng/ml), gp120_BaL_ (250 ng/ml), or AT-2-inactivated HIV-1_Lai_, HIV-1_BaL_, or an HIV-1 clinical isolate (R5 strain as determine by biological assays) for 30 min in binding buffer (PBS with 10% FCS) and then washed with binding buffer. Aliquots of qCD4s exposed to either gp120 or HIV-1 were cultured with 10% FCS containing medium alone or medium containing 10% HI patient serum (pooled from five HIV-1-seropositive subjects). Cells that were cultured in medium without 10% FCS were stained with rabbit anti-gp120 antiserum (ABI) followed by anti-rabbit IgG-FITC (DAKO, Hamburg, Germany) and fixed with 1.0% paraformaldehyde. Cells that were cultured in patient serum-containing medium were stained with the same serum followed by anti-human IgG-FITC. The effects of T22 or Enf were studied by incubating cells with the drugs in binding buffer for 30 min on ice followed by exposure to gp120 or HIV-1 in binding buffer containing the corresponding drug. The exposed cells were washed thoroughly with binding buffer containing the corresponding drug, subsequently cultured in the absence (for T22 experiments) or presence (for Enf experiments) of drug for the indicated times. The percent change in cell-bound gp120 was calculated from MFIs.

### Confocal Microscopy

Purified qCD4s were incubated with gp120 (250 ng/ml) or AT-2-inactivated HIV-1. Immunofluorescence was performed by serial staining with goat anti-CD4 polyclonal Abs (R&D Systems), Cy3-conjugated secondary Abs (Sigma), rabbit anti-gp120 antiserum (ABI), anti-rabbit IgG-FITC (Dako), biotinylated anti-CXCR4 monoclonal Abs (R&D Systems), and streptavidin-Qdot 605 (Life Technologies, Carlsbad, CA), in that order. The cells were then fixed with 4% paraformaldehyde. sICs were visualized by staining purified rCD4s with goat anti-CD4 polyclonal Abs, Cy3-conjugated secondary Abs, biotinylated F(ab′)^2^ anti-human Igs (Life Technologies), and streptavidin-Qdot 525, in that order. Multicolor confocal and DIC images with a 512×512 resolution were acquired using a Zeiss LSM510 system with a Plan-Apochromatic 63×1.4 NA oil immersion DIC objective (Carl Zeiss, Oberkochen, Germany) using multi-track scanning.

### sIC Analysis

For phenotypic analysis, PBMCs purified using Ficoll-Paque were stained with anti-CD3-PerCP, anti-CD4-APC, anti-CD25-PE, anti-CD69-PE, and biotin-F(ab′)^2^ anti-human IgG (BioSource) or biotin-F(ab′)^2^ anti-human IgM (Life Technologies), in that order. After washing, the cells were stained with streptavidin-FITC. PBMCs from an HIV-1-seronegative donor were simultaneously stained as a negative control. For longitudinal analyses of sICs, purified PBMCs were cryopreserved at −80°C, and each sample set from the patient was labeled simultaneously.

### Western Blotting, IgG Purification, and HIV-1 RNA in Situ Hybridization

Whole-cell lysates derived from gp120 (250 ng/ml)- or HIV-1-pulsed qCD4s or rCD4s purified from HIV-1-seropositive or healthy individuals were subjected to SDS-PAGE and were transferred to polyvinylidene difluoride membranes and blotted with antibodies against gp120 (Life Technologies), p24 (Life Technologies), or human-IgG (Dako) after blocking with TBST/5% milk. Proteins were visualized using the SuperSignal West Pico Chemiluminescent Kit (Thermo Fisher Scientific, Waltham, MA) and Biomax-MR film (Kodak, Rochester, NY). IgG purification and flow cytometry-based HIV-1 RNA *in situ* hybridization were performed using the Melon-Gel IgG Spin Purification kit (Thermo Fisher Scientific) and ViroTect (Invirion, Oak Brook IL), respectively.

### Macrophage Ab-dependent Cellular Phagocytosis (ADCP), TUNEL, Fc-OxyBurst, and Chemotaxis Assays

Macrophages were cultured in X-VIVO 10 (Lonza, Zurich, Switzerland) containing 10% heat-inactivated human serum AB (Lonza). Macrophages were collected on day 5 of culture and labeled with Orange-CMTMR (5 nM) (Life Technologies). Target qCD4s were coated with gp120 (250 ng/ml unless otherwise specified) or AT-2-inactivated HIV-1 at 4°C for 1 h, washed thoroughly, and exposed to HI- or non-HI-patient serum for 1 h at 37°C. After labeling with CFSE, qCD4s were incubated with Orange-CMTMR-labeled macrophages (5∶1) in X-VIVO 10 in glass bottom dishes (*Matsunami Glas*s, Osaka, Japan). After fixation, the number of macrophages containing qCD4s was determined from three-dimensional reconstructions generated using an LSM 510 system. The percentage of macrophages that phagocytosed rCD4s was determined using approximately 500 macrophages per experiment. The TUNEL reaction was performed using the FragEL-DNA fragmentation detection kit (Oncogene, La Jolla State, CA). Fc-OxyBurst assays (Life Technologies) were performed according to the manufacturer’s instructions. PBMCs from one HIV-1-seronegative individual were used as the standard for calculating the percent change and relative quantities of oxidative species generated by macrophages as follows: relative O^•^ production = (F_sample_ – F_min-sample_)/(F_ref_ – F_min-ref_) where F_min-sample_ and F_min-ref_ are background MFIs in the patient sample and control, respectively. qCD4 chemotaxis activity was determined using 5-µm microchemotaxis plates (NeuroProbe, Gaithersburg, MD) as described [34].

### Macrophage ADCP Time-lapse Microscopy

For time-lapse microscopy, two-color confocal and DIC images were collected every 30 s with a Zeiss LSM 510 system with a Plan-Neofluar 40×1.3 NA oil immersion DIC objective (Carl Zeiss).

### NK Cell ADCC Assays

Purified qCD4s were coated with gp120 (250 ng/ml unless otherwise specified) or AT-2-treated HIV-1. Highly purified CFSE-labeled (3 nM) NK cells were incubated for 48 h with gp120/HIV-1-coated qCD4s in 10% serum from HIV-1-seropositive subjects, and cytotoxicity was determined by PI labeling (100 µg/ml). Cytokine effects were studied by culturing purified NK cells for 42 h with or without IL-2 (50 ng/ml) or IL-15 (10 ng/ml) in complete medium with 10% FCS. After the cells were labeled with CFSE, ADCC assays with gp120-coated qCD4s were performed.

## Supporting Information

Figure S1 **Rapid turnover of CXCR4 on A3.01 T lymphoma cells and activated qCD4s. (a)** The schematic summarizes the inhibitory activities of the indicated compounds. **(b)** Effect of inhibitors on CXCR4 expression on A3.01 and activated qCD4s. **(c)** After T22 exposure, anti-CXCR4 mAb (12G5) binding to CXCR4 was assessed by FACS. Percent recovery was calculated using MFIs. Bars indicate SD. Data are representative of three independent experiments.
(EPS)

Figure S2 **Turnover of cell-bound gp120 or HIV-1 on qCD4s or anti-CD3 Ab-exposed qCD4s. (a)** Representative FACS data. **(b)** The quantity of cell-bound p24 on HIV-1_Lai_-pulsed qCD4s was assessed by immunoblotting with anti-p24 Abs. qCD4s were pulsed with HIV-1_Lai_ in the presence or absence of Enf. Numbers indicate cell-bound p24 relative to cell-bound p24 at 0 h. Data are representative of three independent experiments.
(EPS)

Figure S3 **HIV-1 patient serum contains sufficient levels of anti-gp120 Abs to form sICs on qCD4s.** Summary of the percentages of Leu3a/CD4v4 (left) and the MFIs of sICs on qCD4s exposed to the indicated concentrations of the gp120 (right). gp120_IIIB_ was incubated at various concentrations with qCD4s, which were then stained with Leu3a and CD4v4 or serum from HIV-1^+^ patients.
(EPS)

Figure S4 **cICs in the serum of viremic HIV-1^+^ Pts are sufficient to form sICs on B cells but not on resting CD4^+^ T cells. (a, b)** Summary of the percentages **(a)** and representative FACS data **(b)** of IgM^+^ or IgG^+^ sICs or IgM^+^ sIC formation on purified CD20^+^ IgG^dull^ IgM^dull^ B cells after exposure to serum from a healthy control donor or HIV-1^+^ Pts with various VLs. **(c, d)** Summary of the percentages **(d)** and representative FACS data **(c)** of fluorescence-based HIV-1 RNA *in situ* hybridization in B cells exposed to serum from a healthy control donor or HIV-1^+^ Pts with various VLs. Plasma VLs are indicated next to the HIV-1^+^ Pt numbers. **(e)** Summary of the percentages of sIg^+^ rCD4s in gp120-pulsed or non-pulsed qCD4s that were exposed to serum **(gp120+serum or Serum)** or the percentages of sIg^+^ rCD4s in non-pulsed qCD4s that were exposed to purified IgG (100 mg/ml) **(IgG)** from a healthy control or HIV-1^+^ Pts with various VLs.
(EPS)

Figure S5 **Time-lapse microscopy of phagocytosis of gp120-coated qCD4s and sIC^+^ qCD4s by macrophages.** **(a, b)** Representative time-lapse image sequence of phagocytosis of gp120-coated qCD4s **(a)** and sIC^+^ qCD4s **(b)** by macrophages. The color overlay images show macrophages (Orange-CMTMR, red) and qCD4s (CFSE, green). Schematic figures and trajectories of qCD4s (various colors) and macrophages (red) are also shown.
(EPS)

Figure S6 **Three-dimensional images of phagocytosis of sIC-coated qCD4s by macrophages.** Data show 3D image reconstruction of deconvoluted stacks through X-Y-Z projections of fluorescence confocal micrographs of phagocytosis assays at 3 h. The color overlay images show macrophages (Orange-CMTMR, red) and qCD4s (CFSE, green).
(EPS)

Table S1 **Percentage of expression of CR and FcγRII in B and CD4^+^ T cells from patients and controls.**
(DOCX)

Movie S1 **Time-lapse microscopy of phagocytosis of gp120-coated qCD4s by macrophages.** The color overlay images show macrophages (Orange-CMTMR, red) and qCD4 (CFSE, green).
(AVI)

Movie S2 **Time-lapse microscopy of phagocytosis of sIC^+^ qCD4s by macrophages.** The color overlay images show macrophages (Orange-CMTMR, red) and qCD4 (CFSE, green).
(AVI)
