# Supplementary material for: Slow Turnover of HIV-1 Receptors on Quiescent CD4+ T Cells Causes Prolonged Surface Retention of gp120 Immune Complexes In Vivo
Source: PLoS One. 2014 Feb 6;9(2):e86479. doi: 10.1371/journal.pone.0086479 (PMC3916329; doi:10.1371/journal.pone.0086479)
Supplement: Table S1 — Percentage of expression of CR and FcγRII in B and CD4+ T cells from patients and controls. (DOCX) [file pone.0086479.s007.docx]

| **Table S1**; Percentage of expression of CR and FcγRII in B and CD4^+^ T cells from patients and controls. | | | | | | |
| --- | --- | --- | --- | --- | --- | --- |
|  | CR1; CD35 | CR2; CD21 | CD11b | CD11c | CD18 | FcγRII; CD32 |
| B cells from healthy donors | 96.2 ± 2.9 | 93.1 ± 4.7 | 82.5 ± 5.8 | 37.1 ± 14.4 | 99.8 ± 0.1 | 96.9 ± 3.3 |
| Activated CD4^+^ T cells (CD3^+^CD4^+^CD25^+^CD69^+^) from healthy donors | 2.6 ± 0.5 | 1.6 ± 0.9 | 3.8 ± 3.1 | 0.5 ± 0.4 | 99.5 ± 0.1 | 0.2 ± 0.1 |
| Resting CD4^+^ T cells (CD3^+^CD4^+^CD25^-^CD69^-^) from healthy donors | 0.5 ± 0.1 | 0.1 ± 0.1 | 1.8 ± 1.1 | 0.1 ± 0.1 | 99.8 ± 0.3 | 0.1 ± 0.1 |
| Activated CD4^+^ T cells (CD3^+^CD4^+^CD25^+^CD69^+^) from Pts | 2.5 ± 1.1 | 0.1 ± 0.1 | 3.4 ± 2.1 | 0.5 ± 0.6 | 99.4 ± 0.1 | 0.2 ± 0.1 |
| Resting CD4^+^ T cells (CD3^+^CD4^+^CD25^-^CD69^-^) from Pts | 0.5 ± 0.1 | 0.1 ± 0.01 | 1.9 ± 0.9 | 0.3 ± 0.2 | 99.5 ± 0.1 | 0.1 ± 0.1 |
| CR3 and CR4 consist of a β-chain (CD18) and an α-chain containing CD11b and CD11c. Pts=patients. | | | | | | |
